# Is it smoking or related lifestyle variables that increase metabolic syndrome risk?

**DOI:** 10.1186/1741-7015-11-196

**Published:** 2013-09-03

**Authors:** Mikael Rabaeus, Patricia Salen, Michel de Lorgeril

**Affiliations:** 1 Clinique La Prairie, Montreux, Switzerland; 2 Laboratoire Cœur et Nutrition, TIMC-IMAG CNRS 5525, Université Joseph Fourier, Faculté de Médecine, Grenoble, France

**Keywords:** Alcohol drinking, Cancer, Cardiovascular disease, Dietary habits, High-density lipoprotein, Lifestyle, Metabolic syndrome, Obesity, Overweight, Physical activity, Smoking, Triglycerides

## Abstract

Metabolic syndrome is considered as mainly caused by a deleterious lifestyle (sedentarity and diet). That smoking contributes to metabolic syndrome had been suggested by several small studies and a meta-analysis. The interesting study by Slagter *et al*. published in *BMC Medicine* is the first very large study confirming this association in both genders, in all classes of body mass index, and in a dose-related manner. Surprisingly, smoking is even associated with increased abdominal fat. Rather than a direct causal effect of smoking, the reason for these associations is most probably the frequent presence of other lifestyle components in smokers. For example, physical inactivity and alcohol drinking are known to be more often present in smokers and could completely explain the observations of the Slagter *et al.* study. Unfortunately, these factors, already not properly checked in the first studies, were not assessed at all in the present one. However, as it is still on-going, we hope that other lifestyle factors will be included in future publications.

Please see related research: http://www.biomedcentral.com/1741-7015/11/195.

## Background

Both smoking and the metabolic syndrome (MetS) - defined as a cluster of biological and physiological characteristics [1,2] - are positively associated with an increased risk of cardiovascular disease (CVD), cancer and all-cause mortality [3-6].

Are these associations causal? One way to answer this question is to determine the possible biological mechanisms that would underlie causality. Such mechanisms, by which chronic smoking results in more cancers and CVD, are fairly established [7,8]. By contrast, the mechanisms behind the association between MetS and either cancers or CVD are much less clear [2]. We do not know whether this association is the consequence of the deleterious lifestyle itself having led to MetS, or of the specific metabolic abnormalities that characterize MetS. For example, is it the lack of physical exercise that is harmful, or the high triglyceride and/or low high-density lipoprotein (HDL) levels resulting from the lack of physical exercise? Importantly, the clinical management of MetS - and therefore the prevention of CVD and cancer - will depend on this understanding of the relationship between MetS and cancer or CVD [2]. In the first hypothesis, prevention would be exclusively based on lifestyle changes. In the second case, physicians would have to concentrate on prescribing drugs - whatever the toxic side-effects [2] - to minimize the various parameters that characterize MetS [2].

## Discussion

One important lifestyle issue is whether smoking by itself may increase the prevalence of MetS. Several studies have shown an association between smoking and some characteristics of MetS: high triglycerides, low HDL, high body mass index (BMI) or abdominal obesity [9-11]. Moreover, in some of these studies, the number of cigarettes smoked per day was linearly associated with the degree of abdominal obesity among smokers; however, no significant difference was observed between smokers and nonsmokers [12]. It must not be forgotten that smoking cessation is known to result in significant weight gain [13], which in turn may, in theory, reduce some of the clinical benefits expected from smoking cessation.

In the context of these quite confusing data, the study by Slagter and colleagues is welcome [14]. Analyzing data from a very large population-based cohort study (about 60,000 people), they report that smoking is associated with a higher risk of MetS in both genders and in all BMI classes. This important finding confirms a previous meta-analysis summarizing data from 13 studies, involving 56,000 participants, in which Sun *et al*. also detected a significant positive association between smoking and risk of MetS [11]. The increased risk was strong for heavy smokers but not significant among light and former smokers, which suggested a kind a dose-effect relationship between smoking and MetS, possibly indicating a causal relationship. There are, however, many limitations in such a meta-analysis with, for instance, various definitions of MetS across studies, racial differences in the study populations and substantial heterogeneity in the way of measuring exposure and cofactors. Thus, analyzing a single large and homogeneous population as done by Slagter and colleagues is very important [14]. All the more so because such a comprehensive and large-scale analysis has not been performed to date.

Slagter *et al*. also investigated the relationship between smoking and the individual components of MetS. The largest differences between smokers and nonsmokers were observed in the levels of HDL and triglycerides, and - to a lesser extent - in waist circumference. This was observed in both men and women, irrespective of their BMI. By contrast, there was no association between smoking and either blood pressure or fasting blood glucose.

Such a kind of dose–response relationship between smoking and MetS components, with the largest effect on HDL and triglycerides, had already been observed in another study, comprising 1,164 men, in those who smoked more than 40 cigarettes per day [9].

One surprising observation in these studies - going against current knowledge - is the positive relationship between smoking and abdominal obesity. It is generally admitted that quitting smoking usually results in weight gain. Smoking is therefore expected to be inversely correlated with weight and BMI. So how can we explain that smoking may increase some forms of obesity? This leads to a fundamental question: is it smoking itself that results in higher risk of MetS or some lifestyle factors associated with smoking?

The overall lifestyle of smokers is generally considered different from the lifestyle of nonsmokers. Accordingly, multiple interactions with other lifestyle factors are expected in smokers. It is likely that the main confusing interaction in the association of smoking with obesity and MetS parameters lies in physical activity. Sure enough, jogging and cycling are rarely the preferred leisure activities of smokers. And indeed, in most people, a lack of exercise results in overweight, high triglycerides and low HDL. Furthermore, as shown by Ortega *et al.*[15], the main factor characterizing so-called metabolically healthy obese individuals (that is, not fulfilling the criteria for MetS) is higher fitness. Even when fitness is taken into account, metabolically healthy obese individuals have a better prognosis than non-obese unhealthy ones. Importantly, fitness was estimated through an exercise test. Estimating physical activity through a questionnaire is very often flawed, as the participants are prone to overestimate their level of physical activity. As an example, in a study by Chen *et al*. [9], physical activity was higher (!) among smokers than in former or nonsmokers. This is all the more surprising because smokers had a lower socioeconomic level, a known risk factor for a sedentary lifestyle.

Smoking is also known to be associated with specific alcohol drinking and eating habits. In some (not all) populations, heavy smokers are also heavy drinkers, a habit that may result in weight gain, high triglycerides and high blood pressure, and high HDL. Here again, the relationship between drinking itself and some MetS parameters are complex as other lifestyle factors - including physical activity again - potentially interact with both smoking and drinking. The relationship between drinking and metabolic parameters usually show U- or J-curve aspects: light and moderate drinkers do have better profiles than nondrinkers whereas heavy drinkers present worse profiles compared with light and moderate drinkers and even nondrinkers. But then, light or moderate drinking is usually associated with healthier lifestyle. Therefore, heavy, moderate and light drinking are not just a question of quantity but also of global lifestyle. It becomes important to differentiate between them when including drinking as a cofactor in the analyses of the association between smoking and MetS. The types of beverages - wine versus others - as well as the way of drinking (binge versus others) are additional confounding factors. So heavy, moderate or light smokers and drinkers do not only have different physical activity habits, but also different dietary habits, both playing a clear role in determining MetS characteristics [16-18]. Finally, socioeconomic variables also interfere with lifestyle [19].

Unfortunately, in most studies analyzing the relationships between smoking and MetS, including the one by Slagter and colleagues [14], these multiple and critical lifestyle factors are not measured and analyzed. This is a major limitation - as underlined by most authors - and precludes drawing definite conclusions regarding the effect of smoking on MetS. However, as the study by Slagter *et al*. is still ongoing, we hope the investigators will be able to analyze these multiple interactions in the future.

## Conclusion

Despite some limitations, the study by Slagter *et al*. provides more evidence that long-term lifestyle changes - including smoking cessation - must be the first-line approach to reduce the prevalence of MetS and of its complications. Indeed, it was recently shown that smoking cessation is associated with a lower risk of CVD complications whatever the effects on metabolic parameters, including weight gain [20]. So, in conclusion, when helping smokers to quit, it is critical to educate them on other aspects of a healthy lifestyle.

## Abbreviations

BMI: Body mass index; CVD: Cardiovascular disease; HDL: High density lipoprotein; MetS: Metabolic syndrome.

## Competing interests

The authors declare that they have no competing interests.

## Authors’ contributions

MR wrote the manuscript, which was then re-read and slightly modified by MdeL and PS. All co-authors read and approved the final version of the manuscript for publication.

## Authors’ information

MR is full-time cardiologist at Clinique La Prairie, Montreux (Switzerland). MdeL is cardiologist and full-time researcher at the School of Medicine of the University of Grenoble (France) and at the French National Centre for Scientific Research (CNRS) in the Life Science Department. PS is nutritionist at the School of Medicine of the University of Grenoble (France).
